# Highly Flexible Precisely Braided Multielectrode Probes and Combinatorics for Future Neuroprostheses

**DOI:** 10.3389/fnins.2019.00613

**Published:** 2019-06-18

**Authors:** Taegyo Kim, Kendall Schmidt, Christopher Deemie, Joanna Wycech, Hualou Liang, Simon F. Giszter

**Affiliations:** ^1^Neurobiology and Anatomy Department, Drexel University College of Medicine, Philadelphia, PA, United States; ^2^School of Biomedical Engineering, Science and Health Systems, Drexel University, Philadelphia, PA, United States

**Keywords:** braid, microelectrodes, neural implants, neural interfaces, neuroprosthesis

## Abstract

The braided multielectrode probe (BMEP) is an ultrafine microwire bundle interwoven into a precise tubular braided structure, which is designed to be used as an invasive neural probe consisting of multiple microelectrodes for electrophysiological neural recording and stimulation. Significant advantages of BMEPs include highly flexible mechanical properties leading to decreased immune responses after chronic implantation in neural tissue and dense recording/stimulation sites (24 channels) within the 100–200 μm diameter. In addition, because BMEPs can be manufactured using various materials in any size and shape without length limitations, they could be expanded to applications in deep central nervous system (CNS) regions as well as peripheral nervous system (PNS) in larger animals and humans. Finally, the 3D topology of wires supports combinatoric rearrangements of wires within braids, and potential neural yield increases. With the newly developed next generation micro braiding machine, we can manufacture more precise and complex microbraid structures. In this article, we describe the new machine and methods, and tests of simulated combinatoric separation methods. We propose various promising BMEP designs and the potential modifications to these designs to create probes suitable for various applications for future neuroprostheses.

## Introduction

Tissue response, yield and longevity of invasive neuroprosthetics continue to be issues limiting both basic and clinical deployment and use of current prosthetic designs. Important factors influence tissue response and longevity, such as mechanical tissue interactions and tissue strain, the size of elements introduced and resulting diffusion barriers. These may all figure as design factors in advances toward successful long term implementation of invasive neuroprosthetics. These factors can plague both electrical and optical methods of recording and stimulation. Various methods have been explored to manage these factors, by both miniaturizing (Szarowski et al., 2003; Seymour and Kipke, 2007; Kozai et al., 2012) and altering compliance of implanted neuroprosthetics (Szarowski et al., 2003; Seymour and Kipke, 2007; Ware et al., 2012; Chamanzar et al., 2014; Sohal et al., 2016). Reducing the size of elements in prosthetics automatically increases mechanical compliance due to the fourth order scaling of compliance with reductions in cylinder radius. The increased compliance comes at the cost of increasing fragility of the elements, and problems of deployment to target tissues. In addition to simple size reductions, materials science suggests novel materials, such as shape memory polymer (SMP; Ware et al., 2012), may further reduce stiffness and increase compliance. Drawn material composites of conventional or SMP materials have been used to create filaments of highly ordered cross sectional structures (Anikeeva et al., 2011; Canales et al., 2015) at fine spatial scales.

Although these are remarkable and highly promising materials and methods, the handling and deployment of resulting probes can be complex. Mesh electrodes have been injected from needles (Liu et al., 2015). Other systems are injected with sewing machine like systems (Hanson et al., 2016). In our group we are exploring use of both ultrafine elements, and construction of 3D textile topologies for braided multi-electrode probes (BMEPs). We use tubular and figured braids as a method to create highly compliant probes composed of many ultrafine wires, or filaments, formed on a mandrel or core. This core, can then aid in insertion but is subsequently removed after implantation, leaving only the braids embedded in tissue. Like meshes, braids gain robustness in handling from the interwoven textile features, but retain high compliance (Liu et al., 2015). Further, braid textile methods support 3D topologies of wire that can be used to vary mechanical compliance, or to order recording sites and wires in pre-specified patterns. Constructing braids on this microscale is technically difficult and developing systems to do this led us to patentable braid plate designs (Giszter et al., 2013). Our initial designs supported classical tubular braids. With these we have shown significant gains in compliance compared to some standard methods (Kim et al., 2013), demonstrated chronic recordings in spinal cord of jumping frogs (Kim et al., 2013), and reduced tissue response in rat cortex (Kim et al., 2019). However, a consideration of recording methods in the literature (Gerstein and Clark, 1964) using multisite wires also suggests novel methods of increasing neural yield per wire in single unit electrophysiology by using multisite wires and combinatoric probe designs. The idea is to combine individual wire sites in different locations to provide unique wire combinations with polytrode pickup capabilities at each location, which can then help to localize and separate units along the length of the probe. Potentially very long probes with high neural yields over their length are feasible. To support these new methods practically with our BMEPs we needed an improved braid plate design. Further, before constructing test probes we also wished to explore the signal separation problem relative to potential probe designs. Preliminary explorations suggested that probe topology and signal processing methods needed to be coadapted.

In developing the new braid plate we also sought to construct a system allowing much longer lead braids suitable for large animal tests and deep placements in central nervous system (CNS), as well as for the various peripheral nervous system (PNS) and myographic application possibilities afforded by the BMEP designs.

## Materials and Methods

### Advanced Designs of a Micro Braiding Machine

#### Goals and Features Desired

We build compliant braided electrodes with a patented custom micro braided machine (MBM). We had two goals in developing a more advanced version of our current patented MBM: (1) improving mechanical and electronic parts for braid manufacture in a more stable, faster and safer way and (2) adding capabilities for producing more sophisticated braid features in order to produce more precise and complex micro tubular braid structures, and braid structures suitable for combinatorics and various other applications in near future. We here call the new version the 2nd generation MBM. Based on our goals, we designed the following new features and advances into the 2nd generation MBM.

#### Mechanical Advances Incorporated

The basic braid plate we patented for ultrafine wire braids comprises several elements defined here and referenced in methods and results below:

*Core holder:* this holds the mandrel or core upon which braiding is applied.
*Movers:* cylinders through which ultrafine wire is threaded in order to braid.
*Shelter slots:* static and mobile “parking” spots for movers during the braid process.
*Circular center plate and shelter slots*: an actuated disk with the inner shelter slots, moving any movers placed in inner shelters along a circular arc.
*Outer plate and shelter slots*: a static collection of shelter slots where movers can be “parked” and moved radially in and out from.
*Radial rack and pinion system:* magnetic and mechanical system that moves movers into and out of different shelters. The centrifugal or “catching” motion uses a magnet to capture the movers. The centripetal motion requires racks to only push the movers inward.

##### Smaller circular center plate with more shelter slots

**FIGURE 1:**
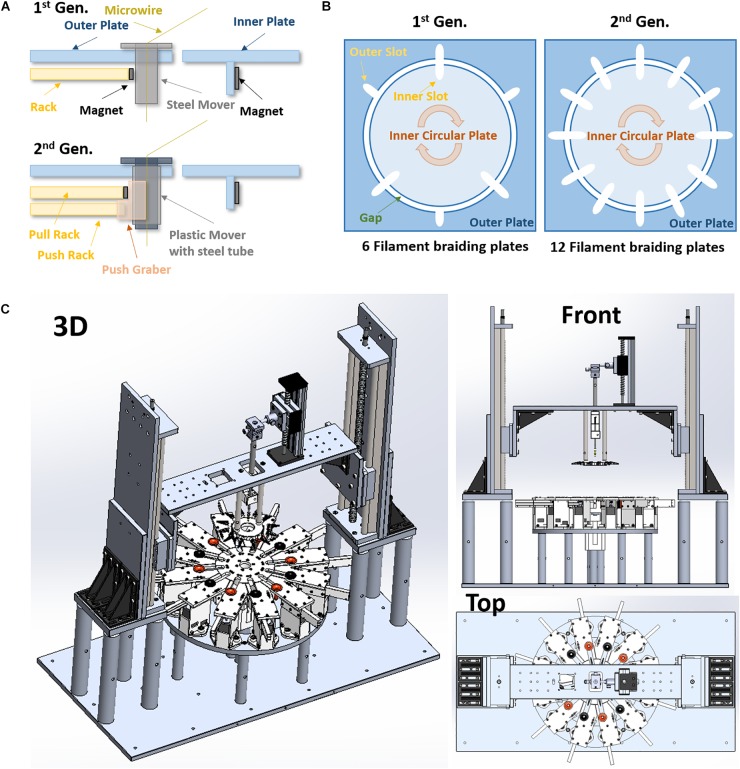
Advanced designs of the 2nd generation micro braiding machine. **(A)** Modified rack and pinion mover driving mechanism (1 rack vs. 2 racks). **(B)** The difference in number of slots for braiding between the 1st and 2nd generations. This is a conceptual diagram to show comparisons. The actual sizes between the 1st and the 2nd generations are different (the both inner and out plates in the 2nd generation are almost half smaller than ones in the 1st generation). **(C)** 3D drawings of the 2nd generation of micro braiding machine in Solidworks.

The circular center plate in the 2nd gen. is smaller than previous designs (diameter 5.2″ vs. 10″), but it has 6 more slots (12 slots in the 2nd gen. vs. 6 slots in the 1st gen., see Figure 1B). Thus, the 2nd gen. MBM can produce more complex 12 single or multi-wire grouped tubular braid structures.

Other advantages of the smaller circular center plate are an increase in braiding speed with a lower linear tangential wire velocity and excursions, less material use and centrally driven load. The tangential moving distance between slots in the circular center plate of 2nd gen. MBM is shorter than that in 1st gen. MBM: the diameter of the 2nd gen. center plate was reduced by almost half. This allows us to increase the speed of the braiding process while remaining at tangential velocities known to be safe for the fragile wires used. The distance between the braiding point on a core and the slot center in the outer plate is a length that is ultimately a leftover wire length, but must unavoidably be added into the total wire material length needed for a specific desired braid length in braiding process. This distance in 2nd gen. MBM is shorter, reducing material that is lost in construction.

To achieve finer pitch, in part already reduced due to the smaller diameter of the 2nd gen. circular center plate, the wire holder plate is constructed so that it can travel and move closer to the circular center plate in the 2nd gen. MBM. A recessed area at the center of the circular center plate is added, designed to prevent the core (extending beyond the bottom of the wire holder plate) from hitting the top surface of the circular center plate.

##### Independent modular outer plates

The outer plate in the 1st gen. MBM is a single square piece having a circular hole at the center for the circular center plate. The 2nd gen. MBM has twelve independent modular outer plates (see Figure 1C). The modular outer plates are easier to assemble, can easily be disassembled for maintenance, and they provide the user with more space for easier access to wires.

##### New rack and pinion design

Rack and pinion mechanisms are the key mechanism to drive movers in and out of slots in the center plate during the braiding process in both the 1st and 2nd gen. MBMs. These differ in the two designs however. The 1st gen design: The small rectangular permanent magnet attached to the inside end of rack toward the center plate was designed to help the narrow rack to push movers held in place with magnets and also to pull movers magnetically out of the center plate. Whenever the racks move backward away from the center plate, movers are pulled by the magnet automatically. To leave movers in the center plate, the circular center plate circular movements served to shear movers along the circular arc and out of the region of magnetic force exerted by the rack magnets before the racks move backward. However, at the moment when the movers release from the magnetic force during the center plate turn, a brief high jerk motion occurs. Micro braiding was possible despite this jerking motion, however, it was not ideal. The 2nd gen. design: The 2nd gen. MBM uses two racks per slot as shown in Figure 1A: one only for pushing the mover with a mechanical grabber without a magnet and the other for pulling the mover with a magnet. Significant jerk in motion does not occur anymore in this design and transitions of movers are much smoother in the 2nd gen. MBM. To drive two racks separately, two stepper motors with potential for microstepping control are used per slot and so a total of 24 stepper motors are required in order to drive the 24 rack mechanisms for the 12 slots in the 2nd gen. MBM.

##### Z-axis and core holder height automation

The 1st gen. MBM did not have any *Z*-axis and core holder height automation. Users had to manually adjust the heights and then the selected heights were fixed during braiding. Thus, the braid pitch continually decreased, albeit slightly, while braiding. The deviations were small with the larger distances of wire due to the larger inner plate diameter. Reducing the inner plate diameter in the 2nd gen. would necessarily increase these pitch changes, absent the new height control. With the added height automation, the 2nd Gen. MBM fabricates a microbraid structure having a consistent and controllable pitch along the braid length by automatically adjusting wire angles, achieved by periodically moving the wire holder plate up with about 0.4 μm resolution. For this *Z*-axis automation, two linear stages with a maximum 40 cm stroke length are used vertically and controlled by two NEMA 23 stepper motors with native 400 steps (0.9° angular accuracy) per one full rotation (see Figure 1C). For the core holder height automation, a mini linear stage is mounted onto the top of the center bridge connected to both linear stages for *Z*-axis automation and controlled by a NEMA 17 stepper motor with 200 regular steps. These three stepper motors are controlled with 1/32 microsteps for high precision rather than full step and thus, in the case with the 400 full step motors, the precision would be 12800 microsteps (0.0025° angular step) per one rotation. This supports long braids and precise braiding pitches.

##### Swappable core holder design

Instead of a fixed core holder design, for the 2nd gen. MBM, we developed a swappable core holder design. This allows users to swap in and out the different core holders necessary for different types of applications requiring cores of varying size, shape, and material. The fixed base holds any one of the magnetically attachable core holders.

##### Adjustable micro position of core holder

Though all mechanical parts used in the 2nd gen. MBM are precisely fabricated by a CNC (Computer Numerical Control) machine shop, in order to compensate any micro-level errors and accommodate the difference of core center positions due to different core sizes and holder variations, a two axes micro-size micromanipulator was used on the top of core holder in the 2nd gen. MBM. This allows initial core centering independent of all other motions actuated.

##### New mover design

Movers used in the 1st Gen. were steel nuts with threaded inner tubes which were filed down to make a smoother inside surface. However, the inner surface was not completely flat, still containing some wrinkles. In the 2nd Gen., an inner 3D printed PLA flanged tube and an outer steel tube were combined to be used as the movers (see Figure 1A). The inner printed tube has smooth curves and a flat inner surface so as to reduce the friction between microwire and the inside surface of the tube in order to protect the wire insulation. Different inner PLA or other material tubes can be swapped in. The outer steel tube still provides good magnetism for the magnetic driving mechanism and allows consistent external mechanics with different inner sleeve designs. The new mover is not only smoother, for the safety of wire, but also lighter weight with current inner sleeves.

##### Reduced gaps and higher tolerances

In the 1st Gen. MBM, the gap between the center plate and the outer plate was 0.25″ (6.25 mm). This was unfortunately large enough for the mover to fall off track occasionally, causing possible damage to the contained wires. For wire safety, the gap was reduced to 1 mm in the 2nd Gen. MBM to prevent any mover falls.

#### Electronic Controls and Advancements

##### New electronic control hardware

The 1st gen. MBM used a general three axes CNC machine control hardware to drive braiding: one axis used to control three stepper motors en masse, as a group for clockwise turns, another one to control three stepper motors as a group for counterclockwise turns, and the other to control the center motor for the circular center plate turns. To fabricate more complex tubular structures and to have the ability to change the path direction of a strand independently during braiding, it is necessary to control each stepper motor independently and run motors in parallel. For this purpose, we developed our own custom electronic control hardware with 28 conventional stepper motor controllers (SMCs), a microcontroller board for I2C serial communication master and an Arduino board for managing and communicating sensor inputs.

##### Expandable stepper motor controller design

Each individual SMC board has a dedicated stepper motor driver and a dedicated microcontroller which handles command sets to control the built-in stepper motor driver and supports various communication protocols such as RS232 serial, I2C serial protocol, etc. In our hardware design, we chose the I2C serial communication protocol to transceive control commands and data among devices. In the I2C protocol, the I2C master board controls serial communications with I2C slaves, each having non-overlapping separate addresses via three shared lines on the I2C bus. In the 2nd gen. MBM, all 28 SMCs work as I2C slaves with such separate addresses and communicate with the I2C master board which finally communicates with a PC via a USB cable. SMC controllers are organized into modules. A total eight SMCs are socketed on a custom printed circuit board (PCB) as a group. These group boards are expandable or stackable via three pin I2C cascading connection of up to 16 boards (for a total of 128 SMCs) – the maximum number of available addresses in the I2C protocol is 128. In the future, the new electronic control hardware can thus easily be expanded for additional functions working with stepper motors by stacking additional 16 SMC group boards.

##### Independent and parallel stepper motor control

Because each SMC is dedicated to each stepper motor used in the 2nd gen. MBM, each stepper motor runs independently of other motors. It also runs in parallel since the SMC executes a control command right after receiving the command. This requires sensory validation of commands to avoid errors. The advantages of independent and parallel stepper motor controls follow below.

###### Individual error correction

In the 1st gen. MBM, the errors of rack length and gap distance could not be corrected electronically since three motors are controlled by one motor driver at the same time. Thus, the only solution was to use a physical compensation method which inserted some small pads behind the racks to adjust the starting position of the racks, depending on error in position. In the 2nd gen. MBM, these errors are easily corrected electronically.

###### Increase in braiding speed

Since SMCs run in parallel and independently, the centrifugal catching rack using the magnet can proceed to advance before the coming mover on its center plate shelter arrives at the corresponding slot, and catch the mover immediately after its arrival. The center plate can also turn immediately after the mover gets out of the center plate shelter and before it gets to the end of its slot and arrives at its shelter in the outer plate. In this way, dead time between different motions is minimized and braiding speed increased.

###### Change in the individual path direction

During the braiding process, all movers are divided into two groups: the clockwise turning group and the counterclockwise turning group. Since SMCs operating in parallel and independently control their corresponding stepper motors, one or multiple movers in the 2nd Gen. MBM can skip a group movement and instead stay at the current slots. This skip can allow the wires in stationary skip movers to trade between groups and reverse their initial direction determined by the clockwise or counterclockwise group. This skipping technique provides the flexibility to control the path direction of individual wires throughout the braids during the braiding process, and create *figured braids*, which was not possible without pausing the machine and applying manual changes in the simpler original design.

###### Expandable digital I/O design

An Arduino board is used to handle a maximum of 40 digital I/Os which could be any kind of 5V digital inputs or outputs (e.g., 5V sensor outputs). This Arduino board is also on the I2C serial bus for the SMC control and so works as an I2C slave. Thus, the number of digital I/O lines could in principle be extended up to 5120 by adding 128 Arduino boards (40 digital I/Os per Arduino) onto the I2C bus.

###### Mover detection sensors

For safety, the 2nd gen. MBM has proximity object detection sensors to check for the presence of movers, placed under the bottom of outer plates. This system is designed to stop the braiding process when an error occurs (see Figure 5D).

###### Custom control software via USB

The electronic control hardware is managed and controlled by a PC regardless of generation. The control software for the 1st gen. MBM was a generic CNC machine program based on the industrial standard G-code. This G-code has a serial execution mechanism which means multiple G-codes cannot be executed in parallel. The control software for the 2nd gen. MBM no longer relies on G-codes because the SMC runs independently and parallelly with their dedicated command sets. For this, we have developed our own Software Development Kit (SDK) to control SMCs via USB-I2C communications and a custom control program for the 2nd gen. MBM based on the developed SDK. The custom control program provides all convenient automatic features for braiding and is extensible through the SDK.

#### Functional Advancements

##### Tubular braiding with maximum 12 individual microstrands

The 1st gen. MBM has six slots in the outer plate and three slots in the center plate, which means the machine can make tubular microbraid structure with a maximum of six individual microstrands. The 2nd gen. MBM has 12 slots in outer plates and 12 slots in the center plate and so it can make tubular microbraid structure with a maximum of 12 individual microstrands, each consisting of single or multi-wires.

##### Changeable braiding pattern

**FIGURE 2:**
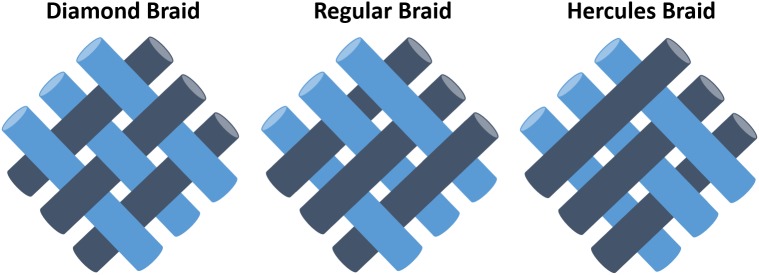
Three common types of braiding structure. Diamond braid: all filaments keep going over and under alternately at every cross point. Regular braid: all filaments go over at a cross point and go under at next consecutive two cross points or vice versa. Hercules braid: all filaments go over at a cross point and go under at next consecutive three cross points or vice versa.

The 1st gen. MBM makes only the tubular diamond braid pattern: 1/1 intersection repeats. The 2nd gen. MBM can make various braid patterns: diamond braid, regular braid (also called plain braid, 2/2 intersection repeat, 2/1 or 3/1 intersection repeat) and Hercules braid (3/3 intersection repeat) as shown in Figure 2. The incorporation of independent stepper motor controls for all shelters allows the system to change braid patterns. Figured braids are possible.

##### Independently changeable braiding direction of individual microstrand

The ability of independent stepper motor control in the 2nd gen. MBM allows control of a single strand out of the currently repeating braid pattern, for “lay-ins” and to form a specific geometry by freely changing the path direction of the selected strand. This feature is mainly useful for (1) combinatorics applications which can increase yields of neural recording via combinations of multiple recording sites on a single wire and precise juxtapositions of multiple recording sites on multiple wires or (2) to lay-in fiberoptic or carbon fiber elements throughout the braid.

##### Automatic braid pitch control

Automatic braid pitch control is realized by *Z*-axis automation with a stepper motor. The main purpose of this feature is to maintain a desired braid pitch during the braiding process in order to fabricate a tubular braid having consistent pitch distance over the whole braid length. This is achieved by periodically adjusting up the wire holder. This holder is mounted under the bridge which is connected to both left and right linear stages that are automatically controlled by stepper motors. This feature is only available in the 2nd gen. MBM.

### Neural Recording Setups Based on Used of New 2nd Gen. BMEPs

**FIGURE 3:**
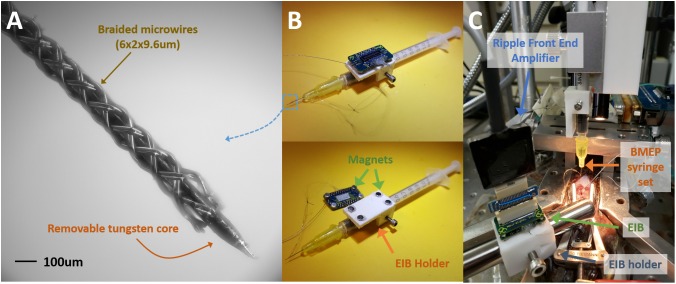
Common BMEP configuration and recording setup typically used with a frog spinal cord recording preparation. **(A)** Zoomed in photo of braided microwires over the sharp tip removable tungsten core. The measured diameter of this braid was 136 μm in the imageJ graphic analyzer tool. **(B)** The BMEP syringe set. The EIB is detachable from the EIB holder and magnetically attached. **(C)** Neural spike recording setup with a spinal frog. The EIB was transferred to another EIB holder fixed on the stainless steel rod from the EIB holder on the syringe set. After implantation the syringe can be removed.

Spinal frogs were prepared under anesthesia in accordance with USDA and PHS guidelines and regulations following IACUC approval. Current BMEPs for neural recording in brains and spinal cords are prepared on a 1 ml syringe set with an electrode interface board (EIB) to make the electrical connections between the electrodes and a recording/stimulation device (see Figure 3B). The sharp tip removable tungsten core (see Figure 3A) is attached to the piston surface inside the syringe. Thus, piston movements cause the tungsten core to move not only in and out of the blunt syringe tip, but also in and out of the braid. After braiding microwires (6 × 2 × 9.6 μm Nichrome wires) over the tungsten core, the braid is trimmed by mechanical cutting to adjust the length of braid and at the same time expose the tips of microwires for recording. All microwires coming out of the other end of braid are connected to individual through-hole contacts on the EIB via clamping with tiny gold pins. We recently switched the EIB from the discontinued Neuralynx EIB-27 Micro to the new Neuralynx EIB-36-QC-Simple which magnetically attaches to a connecting adapter for the frontend amplifier. For this new EIB, we also developed a 3D printed EIB holder with magnets fixed on the syringe set (see the white block in Figure 3B). This EIB is transferred to another EIB holder before inserting the tip of BMEP into neural tissue (see Figure 3C). After insertion, the tungsten core is removed and the whole syringe set is removed for chronic implantation [see Kim et al. (2013) for more details of this mechanism].

For neural spike recording from the spinal cord of bull frog, the anesthetized frog with 5% tricaine (ethyl 3-aminobenzoate methanosulfate; Sigma E10521) was spinalized, decerebrated, and a laminectomy performed to expose the lumbar area of spinal cord [see Hart and Giszter (2010) for surgical details]. The right portion of dura matter from the center vessel of spinal cord was electrically cauterized, carefully cut from rostral to caudal direction and moved to the left side to clearly expose the right side of spinal cord tissue for BMEP penetration. The prepared frog was placed on a frog saddle and fixed by hip and back clamps on an experiment floating stage (see Figure 3C). The BMEP syringe set mounted on a micromanipulator was carefully and precisely positioned over the exposed spinal cord under a microscope and the tip of BMEP was inserted into the spinal cord tissue at laterally 300 μm right from the center of spinal cord and between L2 and L3 roots. Recordings were performed at 400–1200 μm depth with epidermal electrical stimulation at the ankle of the left hind limb to evoke a wipe movement of the opposite right hind limb. To record neural spikes, we used the Ripple Grapevine Neural Interface Processor with the Nano 2+Stim, stimulation and recording front end.

### Electroplating Setups

Once a BMEP syringe set is manufactured, electroplating of the BMEP recoding sites is required to lower impedance to suitable neural recording levels. Before electroplating, the impedance range of each channel is 2–4 MΩ with 9.6 μm wire and 2–3 MΩ with 12.7 μm wire. Using a custom in-house Matlab program and NanoZ (64 channels electroplating device, by Neuralynx), electroplating is automatically performed to lower the impedance of all individual electrodes of BMEP down to 200 kΩ for recording channels and 50 kΩ for the reference channel. Using a solution of gold solution and multi-walled carbon nanotubes, described previously (Kim et al., 2013), it was not possible to lower impedance to this 50 kΩ. However, using the platinum nanograss coating technique described in Boehler et al. (2015) [2.5 mM Chloroplatinic acid (H_2_PtCl_6_) + 1.5 mM formic acid (HCOOH)], we can achieve impedance levels as low as 20 kΩ without any short circuit issues.

### Computational Simulation for Combinatorics

Simulations of braid based combinatorics signal separation were performed in Matlab. We used the Makeig EEGLab implementations of Infomax Independent Component Analysis (ICA) and built in principal component analysis (PCA) Matlab functions after extraction of single units from simulated probes with mixed signals, using a thresholding method. In addition, we exported simulated but partly processed signals as NEV files using adaptations of Matlab code provided by Ripple, for import into commercial offline spike sorters. As an offline sorter in this paper we employed the Plexon Offline Sorter (POS) for spike separation after spike packet assignment to sites.

#### Combinatorics Choices

**FIGURE 4:**
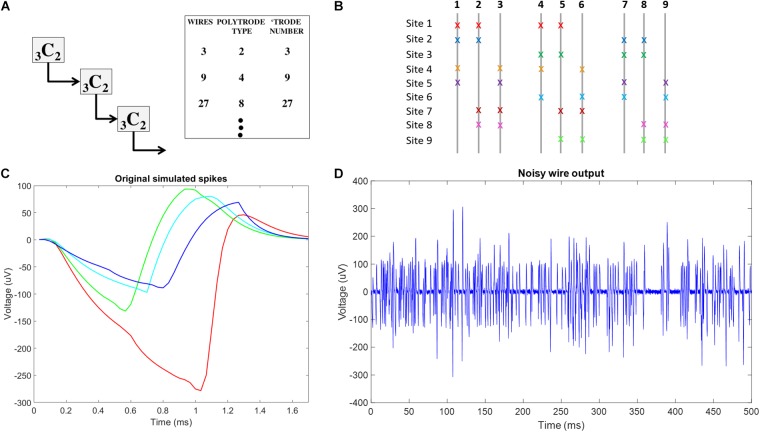
Conceptual schematic for combinatorics choices and neural signal plots used in simulation f **(A)** Schematic of reiterated arrangement in a multiscale approach, where the correspondence between number of spatial sources and number of wires is maintained for the use of ICA in Blind Source Separation. This only uses a subset of the potential number of unique tetrode sites in a _9_C_4_ braiding. **(B)** Combinatorics concept-_9_C_4_ probe design color coded x’s indicate the wires involved at each recording site in the multiscale ICA compliant design. **(C)** The original 4 neural spikes used in this simulation. **(D)** Example of final noisy wire output, showing activity from 16 neurons across 4 recording sites with added gaussian noise on one of the 9 wires in the simulated probe.

Exposing multiple recording sites on a single wire would allow neural activity to be recorded at each site, and the activity from each site would sum together to produce the observed wire output (Gerstein and Clark, 1964). Since, in a standard tetrode grouping (see Gray et al., 1995), or a single wire, the yield of units per wire is usually on the order of 1.3 units, this would increase the number of units recorded from this single wire by 1.3 × S, where S is the number of recording sites. Unfortunately, there is no way to determine from which site specific and unique spikes arise on a multi-site single wire standalone probe. The geometry of BMEPs, however, allows for wires to be manipulated in patterns, of 3D topology, such that specific wires can be placed together at specific sites. This opens the possibility of having multiple recording sites on single wires. These have been used by others, albeit rarely (Gerstein and Clark, 1964). In a multi-site combinatoric BMEP design, by permuting groups of wires, any activity recognized on a single wire with multiple sensing sites, is grouped with the information from other wires, and unmixing activity from each site becomes possible. ICA, a blind source separation algorithm, can unmix linearly combined, independent sources (Bell and Sejnowski, 1995). ICA has proven successful as a spike separation algorithm, as long as the number of recorded spikes is less than or equal to the number of recording sites (Brown et al., 2001). Here we extend this work to separate site level simulated neural activity. Since ICA produces as many independent components (ICs), representing independent sources, as the vector length of the mixtures presented to the algorithm, if the number of mixtures in the vector, in this case, the number of wires, matches the number of total polytrode sites on the multi-site combinatorics probe, ICA can be used to extract spatial site activity from the ensemble data, localizing neurons to spatial sites. This presents an immediate design constraint on building figured braids for spike separation, and the site placements and numbers of sites per wire. Rather than using any arbitrary combination code for sites, the site number and wire number should match, with appropriate balanced numbers of wires at sites. The simplest example of this arrangement is 3 wires arranged such that there are 2 wires per site creating _3_C_2_ = 3 total sites. This yields 3 × 2 × 1.3 = 7.8 units on 3 wires vs. 3.9 in conventional single site recording. This arrangement can easily be scaled up by repeating the _3_C_2_ arrangement, using wire duos, from 3 initial _3_C_2_ sets, instead of individual wires, as base components (Figure 4A). In theory, this arrangement can be repeated indefinitely, however, in practice there is likely a limit to the number of sites per wire that can be used to pick up reliable neural signals due to electrical summing, shunting, and signal event collisions that cannot be easily separated. For demonstration purposes, here we test signal separations in simulation, employing the _9_C_4_ arrangement (Figure 4B), creating a total of 9 recording sites with 4 wires present at each site, yielding 9 × 4 × 1.3 = 46.8 units vs. 11.7 units in conventional single site recording, to conduct a simulation experiment.

#### Simulation Method

For each simulation trial, at each site, we simulated 5 s of 40 Hz Poisson distributed spike trains coming from four neurons with unique spike shapes, assuming a 30 KHz sampling rate. To reflect the refractory period, we ensured that Poisson generated spikes of any simulated neuron were separated by 1.7 ms, the length of our simulated spike shapes. Fifty-two point Spike shapes were generated by modifying a smoothed sawtooth waveform. To simulate slight variations in distance from each wire at each site to each of the four simulated spikes at each site, slight amplitude adjustments were made to spike shapes on each wire. An example of the four unique spike shapes at a single site on a single wire is shown in Figure 4C. The total number of spike trains was thus (9 sites) × (4 wires per site) × (4 neurons per site) = 144. The randn function in Matlab was used to generate a vector of normally distributed random numbers that were then added to each of the 144 simulated spike trains to simulate noise processes at each exposed site on each wire. The 9 wire outputs were generated by summing the 16 spike trains recorded on each wire before adding white gaussian noise, using the Matlab function awgn, with a signal to noise ratio of 4. An example of noisy wire output can be seen in Figure 4D. In total, 10 trials were conducted.

To separate site level activity, the noisy wire output signals were run through the ICA algorithm. Since the 9 wire outputs are effectively linear combinations of the neural activity occurring at the 9 spatial recording sites, ICA should be able to discern the individual site activity and the ICs output by the algorithm were assumed to each represent the activity from one site. The correlation value between each IC and each of the non-noisy simulated site activity trains were computed, and the maximum correlation value was presumed to be the site which that IC represented. Average maximum correlation values were computed over data generated in all 10 trials.

To sort individual unit activity at each site, we used two approaches: (1) Density based spatial clustering of applications with noise (DBSCAN), a density based clustering algorithm first described in Ester et al. (1996) and implemented in the DBSCAN code on Matlab file exchange. (2) Plexon POS using K-Means or contour results as templates. Both approaches were performed on spikes extracted from the ICs of 10 simulation trials using a thresholding extraction method. Evaluation of each method included: determining correlation values between all 36 simulated neural spikes trains (4 neurons at each of 9 sites) and corresponding reconstructed spike trains; calculating hits, measured as the number of reconstructed spikes within a 30 point window of the onset of each simulated spike; misses, measured as the number of reconstructed spikes missing from a 30 point window around the onset of each simulated spike; and false positives, measured as a lack of simulated spike existing within a 30 point window of a reconstructed spike. Results are reported as the averages across all 10 trials.

The DBSCAN method clusters the data by including neighboring data points within a user defined radius, epsilon, from any data currently in the cluster. If the cluster includes less data points than a user specified value, minpts, the cluster will not be considered unique. For simulations conducted here, DBSCAN was performed on the first five principal components of extracted spike data, with starting epsilon = 7 and minpts = 50. A custom Matlab script was written to adjust epsilon by 0.5 until the number of clusters recognized by DBSCAN was 4, the known number of simulated spikes per site. Points not included in clusters are considered noise by DBSCAN, and are discarded from further analysis. Noise here causing failure is due to spike collisions.

Unlike DBSCAN, the K-Means algorithm implementation in Matlab will assign every data point to a cluster, including the noise. POS, in contrast, has a built-in discriminator to omit outliers from K-Means generated clusters. Here, we observed that this feature of POS effectively omits spike collisions from clusters. Data imported into POS was analyzed by channel (site) with the 4 starting K-Means centroids chosen by visual identification of the feature space (principle components 1 and 2). If K-Means failed to identify obvious clusters, the contour feature was used to ensure all 4 clusters were recognized. With spike collisions still being omitted at this point, the 4 identified spike shapes were used to create unit templates, and template sorting was conducted. Since all remaining data were created by spike collisions, shape features were shared with one or more of the templates, and all data was assigned to a cluster.

## Results

### Development of the New Micro Braiding Machine

**FIGURE 5:**
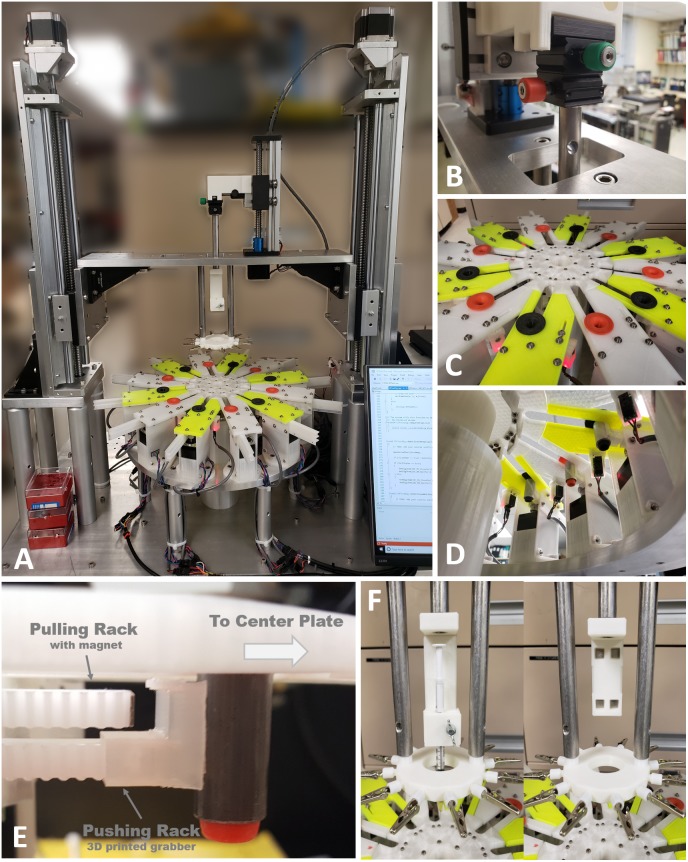
The 2nd generation of micro braiding machine. **(A)** Full view photograph of the micro braiding machine. Behind the LCD touch screen control, which is partially visible at the bottom right corner, the newly developed electronic control box is located (see Figure 4 for details). **(B)** A small micromanipulator is used to adjust the center position of the core holder base. Adjustments center the core depending on different types of core holders used for different core materials and shapes. **(C)** Photo of inner and outer braiding plates with two different colored movers in orange and black. Outer modular plates have two different colors to easily distinguish the two groups used for a traditional simple braid: the clockwise and counterclockwise groups. Red lit tiny white blocks under the outer modular plates are the boxes having object detecting sensors with red LED indicating the status of object detection. **(D)** Photo of the view from under the braid plate. The rectangular black boxes connected with wire bundles seen are the object detecting sensors. (The sensor is inside the small box glowing red light under the front outer plate. Left photo: the red light means that an object (mover) is detected.) The small white rectangular blocks under the inner circular plate are the magnet holders used for centrifugal motions to magnetically grab movers when movers are in the inner central shelters. A plastic tube covers the center motor, wires, and supporting mechanical parts to prevent wires tangling or trapping in moving elements. **(E)** Image of two racks with different tips (magnet and plastic grabber) and a mover from under one of the outer plates, to show the mover driving mechanism as conceptually shown in Figure 1A. **(F)** Photos of detachable core holder system. The left shows the 1 ml syringe holder and the right shows the core holder base with four magnets without any type of core holder.

**FIGURE 6:**
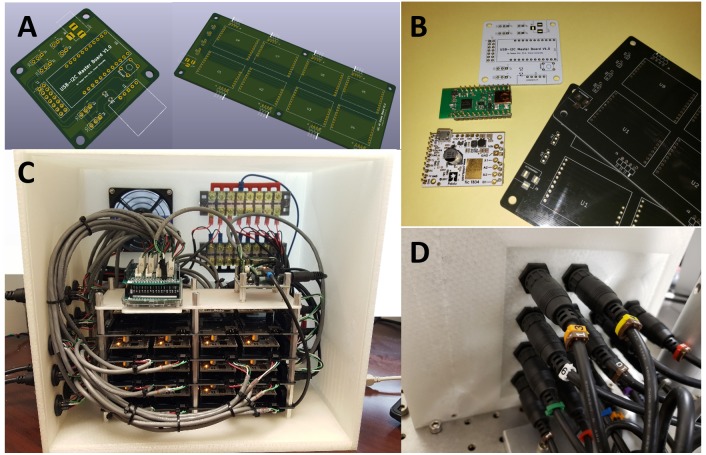
The newly developed electronic braid controls. **(A)** PCB (printed circuit board) designs for the I2C master (left) and the stackable I2C 8 slaves group board (right). **(B)** Manufactured PCBs with components: the white (master) and the blacks (8-slaves group boards) and electronic modules: I2C master controller (green) and stepper motor controller (white). **(C)** Fully assembled electronic devices in a 3D printed enclosure. **(D)** Durable intermediate connectors were installed on the left side of the enclosure for easy connection and disconnection of the many wire bundles.

We successfully built the 2nd generation MBM based on advanced designs described in materials and methods. Figure 5 shows the fully assembled and working MBM. We combined custom aluminum parts fabricated by a machine shop and purchased commercial mechanical parts with plastic parts printed by our own 3D printer. Use of 3D printed parts allowed us to rapidly test the proof of concepts with printed prototypes and easily fix issues with low expense. Figure 6 shows the fully assembled electronic control device for the new MBM in a 3D printed enclosure. It was designed to show working conditions of individual modules with LEDs through the acrylic window in front of the enclosure. Total 4 slave group boards were used to install 28 SMCs (24 for 12 slots, 1 for the center plate, 1 for the small linear stage, 2 for both left and right large linear stage) and one microcontroller was used as a I2C master (see the right module on the most top board in Figure 6C). To check states of 14 sensors and switches (12 object detecting sensors and 2 limit switches for small and large linear stage), one Arduino board was used (see the left module on the most top board in Figure 6C). To easily connect and disconnect multiple wire bundles, intermediate cables having two different types of connectors at both ends were used between the stepper motors and sensors and the control box. We have developed a Windows program to control the MBM via USB communication to the electronic control box and provide users ease of use with the graphic user interface on the touch screen monitor. With this program, users can easily change braiding patterns, braiding speed, the number of filaments in a braid, the length of braid, braid pitch, and can check current states of individual stepper motors, sensors and limit switches, and turn on/off or reset SMCs. Soon we will add a programmable feature for sequencing automation of complex figured braids to this program.

### Micro Braid Samples

**FIGURE 7:**
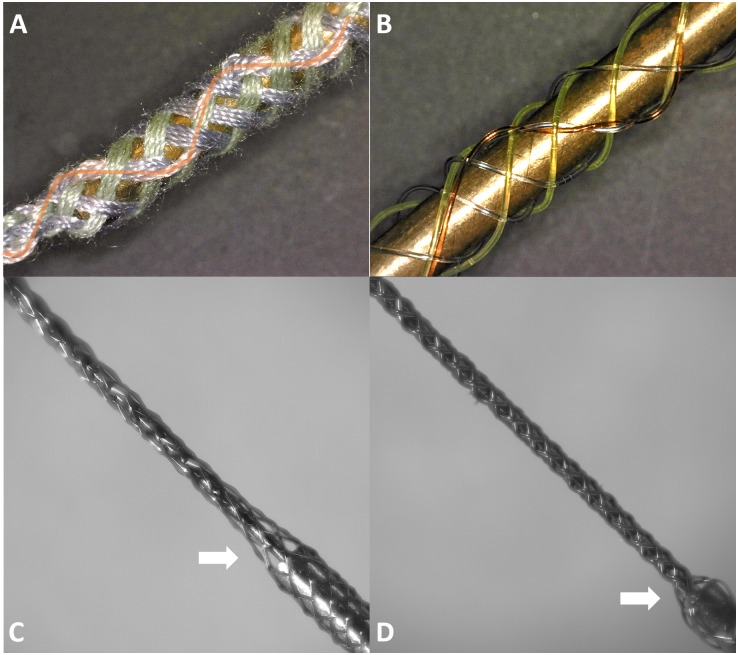
Braid samples fabricated by the 2nd generation of micro braiding machine. **(A)** Test figured patterns: Figured braid sample with cotton threads (7 × 4). Four pink threads have the zigzag pattern on the top side (orange curved line clearly shows the zigzag pattern) while the green threads (3 × 4) and the purple threads (3 × 4) have the typical clockwise and counterclockwise helical pathways in a standard tubular braid. **(B)** Test patterns: Another figured braid sample, with colored nylon fishing lines. **(C)** Final braided microwires (12 × 1 × 9.6 μm) over the tungsten core and a partial portion of blunt syringe tip. **(D)** Final braided microwires (6 × 2 × 9.6 μm) over the tungsten core and a partial portion of blunt syringe tip.

To utilize and test new features of the new MBM, we focused on making BMEPs consisting of single 12 filaments and figured braids, which are tubular braid structures we could not produce with the previous MBM. At first, we tried to make figured braids having a filament that changes its braiding directions multiple times to form a zigzag pattern during braiding. For easy demonstration, we used cotton threads and thick fishing lines to make the figured braids. We successfully made figured braids as shown in Figure 7A,B. In Figure 7A, total 7 groups having 4 cotton threads per group were braided together. Six groups (3 green and 3 purple) have regular braid patterns, clockwise or counterclockwise helical pathways and the 7th group (pink) has a zigzag pattern as we expected while being interwoven in the regular braid of 6 groups. In Figure 7B, the same type of figured braid was manufactured again with 7 thick fishing lines to more clearly show the zigzag pattern (the red line). Next, we succeeded in manufacture of a BMEP consisting of single 12 × 9.6 μm wires as shown in Figure 7C. Interestingly the diameter of BMEP with single 12 × 9.6 um wires is larger than the BMEP with 6 pairs × 9.6 μm wires though both BMEPs comprise 12 same wires in number. This result was consistent with multiple loose or tight BMEPs with single 12 × 9.6 μm wires in different pitches. Another interesting point is that the BMEP with single 12 × 9.6 μm wires cannot follow the sharp changes in 3D contour of a core while the BMEP with 6 pairs × 9.6 μm wires can completely and tightly wrap the core following its 3D shape (compare Figure 7C,D, especially the areas where the two white arrows point). The tubular braid structure of single 12 filaments seems to have a tendency to more strongly maintain the tubular braid structure itself or resist being collapsed. We will talk more about advantages and disadvantages of this property in the discussion.

### Neural Recording

**FIGURE 8:**
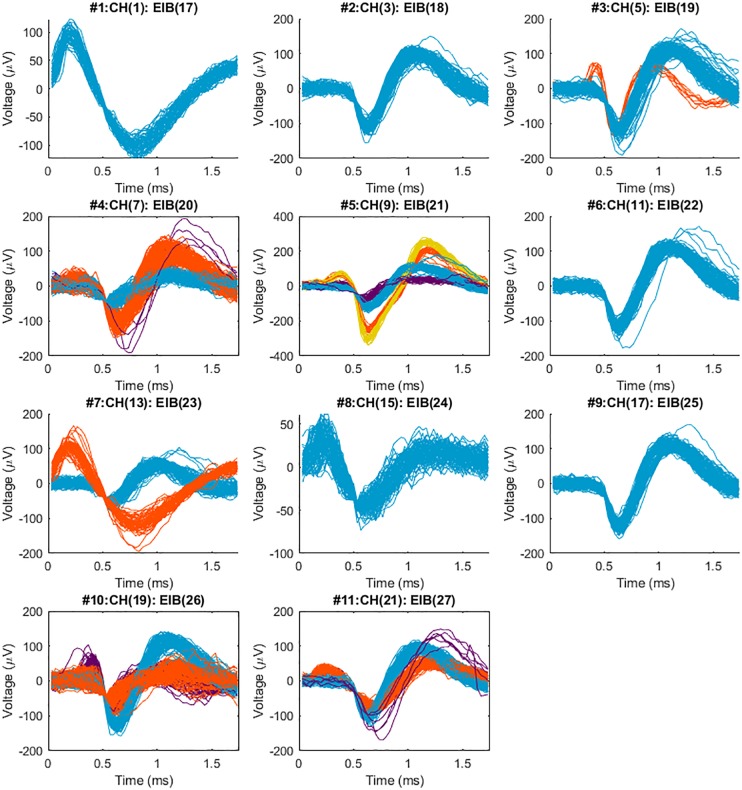
Sorted neural spikes recorded from the spinal cord of a spinal bull frog collected with a 2nd generation MBM constructed BMEP. The BMEP used for this recording consisted of 6 × 2 × 9.6 μm wires (11 recording channels and 1 reference channel) constructed in the new braid system. The recording time was 60 s per trial. Five trials which were recorded at the location and depth (right lateral 300 μm from the center, caudal 800 μm from the L2 root, the 400 μm depth) were concatenated in time series with 60 s time difference between trials and sorted with T-Dist E-M scan method in POS v3.3.5. Above spike waveforms were imported and visualized in Matlab after being sorted in the POS. The maximum SNR (signal to noise ratio) was 38.9 in #5 (the average of yellow spikes), the minimum SNR was 5.2 in #8, average SNR across 11 channels was 14.5 and standard deviation of SNR across 11 channels was 8.0. A maximum of 4 different shapes of neural spikes was observed per channel (in #5) and 2–3 different shapes of neural spikes were easily observed per channel.

As we expected, the different braid structures did not affect the quality of recording performance. Recordings were concatenated in time series and sorted with the T-Dist E-M scan method in the POS v3.3.5. Spike waveforms were then imported and examined and visualized in Matlab. Figure 8 shows sorted neural spikes in 11 channels recorded from the spinal cord of a bull frog with the new BMEPs manufactured by the new MBM. All 11 channels show at least one type of neural spikes and in many channels, 2∼4 different types of neural spikes per channel are easily observed. Surprisingly the highest SNR was 38.9 in #5 channel [the peak-to-peak voltage of yellow spikes is over 600 μV (±300 μV)] and the average SNR across channels was 14.7 ± 4. Even after removing the highest two SNR values by orange and yellow spikes in #5, the next highest SNR value was 20.4 in #4 channel. We have never seen this high SNR with BMEPs in our previous neural recording device before. We think that this is the result of a very low noise floor (smaller than ±30 μV vs. slightly larger than ±50 μV in the previous recording device) and improved SNR of Ripple Grapevine NIP working with our BMEPs compared to older testing systems, and the improved quality of the electroplating with platinum nanograss.

### Combinatorics Processing for More Complex Arrangements of Braid Sites and Braid Design

**FIGURE 9:**
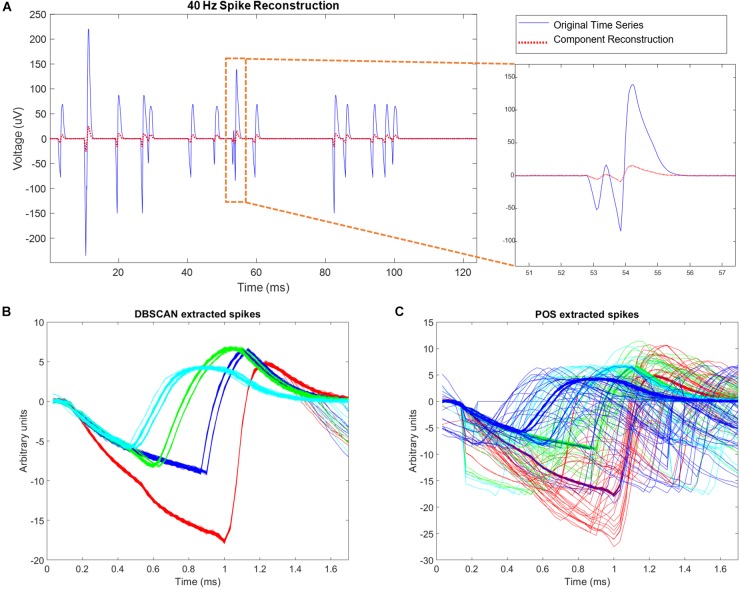
Simulation results. **(A)** Left hand side of the figure shows simulated site activity without noise added in blue, and independent components derived from noisy wire outputs in red. The four different spike shapes can be detected in this figure. On the right hand side of the figure, a site level collision is observed, and preserved by ICA. The activation scale and signal scale differ but the correlation coefficient of the reconstruction is 0.99. **(B)** DBSCAN method showing recovery of only clean non-colliding spikes. **(C)** Plexon Offline Sorter (POS) extracted spikes showing variation in spikes shapes caused by presence of spike collisions that are being assigned to spike clusters.

Using simulations of multisite wires in arrangements compatible with the new 2nd Gen MBM we found good combinatoric signal extraction and yields from 9 wire probes arranged as multisite wires (4 per wire) in an iterated _3_C_2_ pattern. ICA separated each spatial site’s activity nearly perfectly, with correlation values between the original site activity without noise added, and activity reconstructed via ICs averaging 0.9981 ± 0.0007 (Figure 9A). With all spike trains occurring at 40 Hz, collisions of spikes are inevitable, both at the site level, when one or more of the neurons at each site fire within 1.7 ms of each other, or at the wire level, when spikes at different sites occur within 1.7 ms of each other. ICA can unmix wire level spike collisions, reconstructing site activity without evidence of collisions from other sites. ICA also preserves site level collisions, as in Figure 9A.

Correlation values between simulated neural spike trains and reconstructed spike trains based on DBSCAN averaged 0.8276 ± 0.0502, percent hits averaged 69.38 ± 7.287, percent misses averaged 30.62 ± 7.287, and percent false positives averaged 0.7032 ± 4.819. Correlation values between simulated neural spike trains and reconstructed spike trains based on POS Offline sorter use averaged 0.8405 ± 0.07235, percent hits averaged 82.51 ± 7.578, percent misses averaged 17.49 ± 7.287, and percent false positives averaged 5.332 ± 5.951. POS significantly improved the correlation of simulated signal to reconstructed signal (*p* = 0.0022, two sample *t* test). POS also significantly increased the percentage of hits (*p* = 3.7171e-96, two sample *t* test), and significantly decreased the number of misses (*p* = 3.7171e-96, two sample *t* test) compared to DBSCAN. However, the number of false positives was increased significantly (*p* = 8.2566e-43, two sample *t* test) in POS compared to DBSCAN. The false positives metric is a measure of the number of neurons being wrongly assigned to another classified neuron. Since brain computer interfaces may largely depend on the timing of specific neurons spiking to carry out actions, minimizing false positives is very important. The difference in performance of the two methods is evident when observing extracted spikes. Figure 9B shows the pure spike waveforms, free of collisions extracted by DBSCAN, while Figure 9C shows that POS assigns spike collisions to clusters, resulting in a much noisier result. Though the significant increase of hits and decrease in misses in POS compared to DBSCAN seems very positive, the significant increase in false positives obtained by POS should not be ignored, and it is not clear which method would prove best in practice. Likely, this would depend on the application of the sorted data.

## Discussion

### Braids Support Various Possible Designs

BMEPs have significant potential because they are not only mechanically highly flexible microprobes causing less immune-responses in neural tissue (Kim et al., 2019), but can also be easily modified into various designs due to three unique properties: usability of any kind of core, changeable braid patterns with various materials and no length limitation. We will discuss these properties in the following subsections.

#### Braids Are Possible Over Any Kind of Core

**FIGURE 10:**
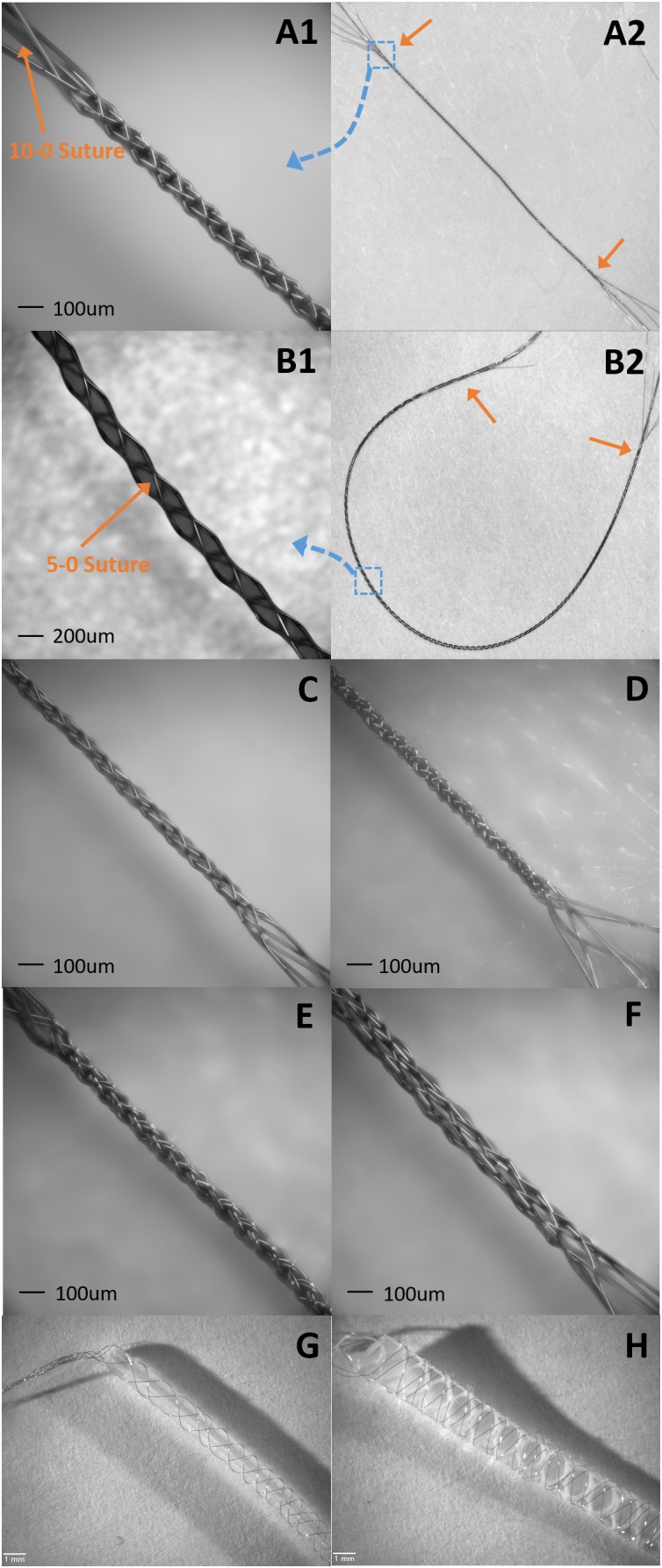
Samples of suture like BMEPs and BMEPs with various cores. **(A1,A2)** BMEP (6 × 1 × 9.6 μm) over a 10-0 suture. **(A1)** The zoomed-in photo in the area of the blue dotted square box in A2. BMEP D_out_ (measured outer diameter) = 99 μm. **(A2)** The full shot of BMEP over 10-0 suture. The length of BMEP between two orange arrows is 1.4 cm. **(B1,B2)** BMEP (6 × 1 × 9.6 μm) over a 5-0 suture. **(B1)** The zoomed-in photo in the area of blue dotted square box in B2. BMEP D_out_ = 200 μm. **(B2)** The full shot of BMEP over 5-0 suture. The length of BMEP between two orange arrows is 4 cm. **(C)** Loose BMEP (6 × 1 × 9.6 μm) without any core. BMEP D_out_ = 66 μm. **(D)** Tight BMEP (6 × 9.6 μm) without any core. BMEP D_out_ = 69 μm. **(E)** BMEP (6 × 2 × 9.6 μm) without any core. BMEP D_out_ = 93 μm. **(F)** BMEP (12 × 1 × 9.6 μm) without any core. BMEP D_out_ = 129 μm. **(G)** BMEP (6 × 1 × 12.7 μm) with a plastic rod. **(H)** BMEP (6 × 1 × 12.7 μm) with a silicone sheet.

Because ultrafine microwires can easily follow and wrap the 3D shape of a core during tubular braiding, any size, shape, and material can be used as a core inside the BMEP. Cores can be removable, dissolvable, or remain in place in BMEPs. Figure 10 shows multiple samples of BMEPs with various cores and without a core. Beside the samples shown in Figure 10, BMEPs over optical fiber and micropipette are possible designs for specific applications, though the innate advantage of mechanical flexibility of BMEPs is reduced due to the higher stiffness of optical fiber and glass micropipettes. If a slowly dissolvable material mixed with a chemical drug, such as an anti-inflammatory agent, is used as a core in a solid cylindrical form in a BMEP, a gradual drug delivery over the whole braid length would be feasible. This might be used to reduce early stage inflammation induced by initial penetration while the BMEP provides electrophysiological functions. In this example, any kind of drug could be used for other purposes.

#### Figured (Varied) Braid Patterns Are Possible Combining Various Materials

**FIGURE 11:**
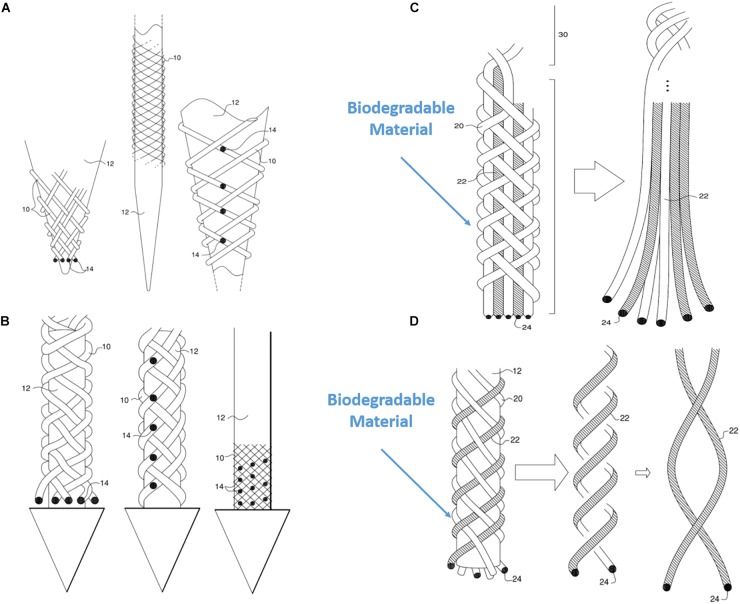
Various possible BMEP designs. **(A)** Variable recording/stimulation sites at tips and on the body over a sharp tip rod or micropipette. Black dots represent the recording or stimulation sites. **(B)** Variable recording/stimulation sites with a protective arrow head to protect tips of braid during insertion and provide easier penetration. **(C)** Example of using biodegradable filaments to wrap wire bundles which are the microelectrodes. After insertion into neural tissue, the straight wire bundle unwrap gradually because the wrapping filaments are degraded in the tissue. **(D)** Example of using biodegradable filaments (white) with microwires (hatched) braided together. Only the microwires in a loose helical form are left in the tissue after biodegradation of the other (white) filaments which are completely dissolved. These drawings are from our US patent (Giszter, 2016).

Another unique property of BMEP designs is the ability to mix various wires and filaments comprising different materials in a single braid. For example, 6 wires individually using two different metals (Pt and Nichrome) could be braided together: Pt for stimulation channels and Nichrome for recording channels. Furthermore, if biodegradable filaments are used to form a braid with other microwires, the initial braid structure could be transformed into a different form after the filaments are completely dissolved in tissue. Figure 11C,D show examples. In Figure 11C, the braid consisting of biodegradable filaments releases the inner wire bundles after the braid is dissolved. In this case, the braid is a temporary wrapper and the inner wire bundles, actual microelectrodes, naturally spread out in tissue. In example of Figure 11D, the braid consisting of a combination of microwires and biodegradable filaments loses its tubular braid structure after being dissolved, and loosely twisted microwires are left and work as microelectrodes. These are just two examples of possible design ideas with biodegradable materials. In another variation of the same braid structure, we can make different combinations of various positions of recording/stimulation sites on the microwire bodies. Figure 11A,B show these examples. If recording sites are located vertically at multiple different positions along with the braid axis, different neural activities at several depths could be recorded at once with a BMEP without moving the BMEP up and down. This technique also allows us to implement combinatoric applications requiring multiple recording sites on a single microwire.

#### Length Limitation Reduced

The new MBM can lift the center bridge holding microwires and the core holder from the lowest level up to 40 cm, which means that the possible maximum length of BMEP is 40 cm with this machine. This length limitation comes from the maximum stroke travel length of *Z*-axis linear stages used in the 2nd gen. MBM. With a longer version of linear stage, we can manufacture BMEPs much longer than 40 cm. Theoretically there is no length limitation if very long continuous wires are used. This opens other options with BMEPs for applications requiring long electrodes or a long spring-like tether for large animals such as primates and for human subjects.

### Preliminary Laser Tests for Insulation Ablation and Cutting

**FIGURE 12:**
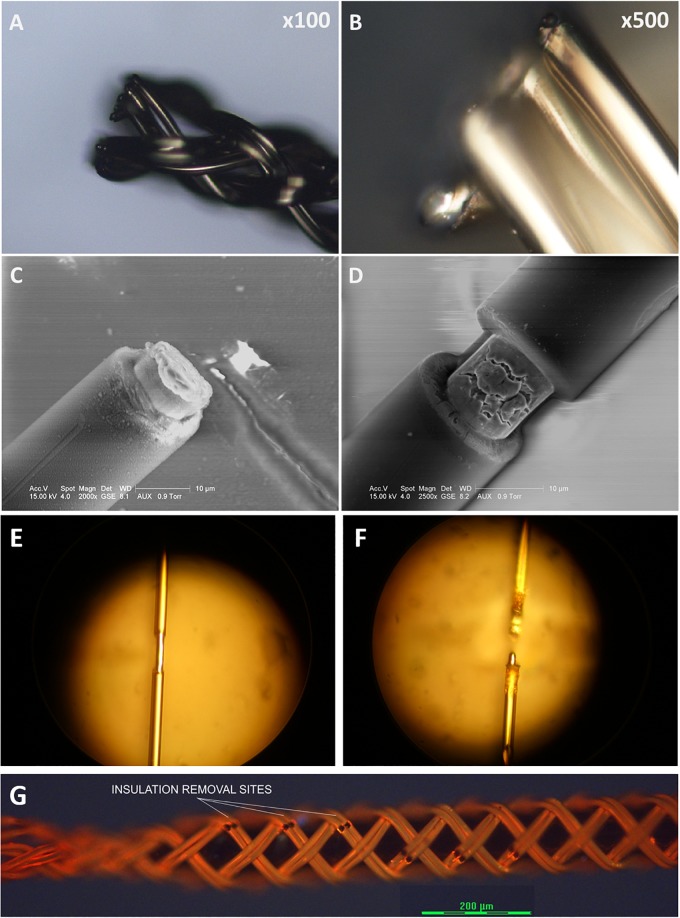
Sample images from various preliminary laser tests for cutting and for insulation ablation needed for precise site placements on a figure braid designed for combinatorics. **(A)** A cutting test on a BMEP (6 × 3 × 12.7 μm) with the line beam of Excimer laser. **(B)** The zoomed-in photo of **(A)**. **(C)** A cutting test on a single 12.7 μm wire with the line beam of Excimer laser. **(D)** An insulation ablation test on a single 12.7 μm wire with the line beam of Excimer laser. **(E)** Insulation ablation on single 12.7 μm wire with 1W UV laser. **(F)** The cutting test on single 12.7 μm wire with 1W UV laser. **(G)** Multiple precise insulation ablation tests on an actual BMEP (6 × 2 × 9.6 μm) sample achieved with the Leica LMD7, laser microdissection system. Black gaps/dots are the insulation removal sites.

To expose a recording/stimulation site on the body of an ultrafine microwire such as 9.6 μm wire (see Figure 12A,B), i.e., not limited to the tip of wire, a laser system is required because the ultrafine wire is too small and fragile to mechanically remove the insulating material in a micro-spot size. It would be also useful if a laser system can cut the ultrafine microwires because mechanical cutting always exerts physical compression forces onto the braid structure and can collapse the braid structure. We have explored many ways to utilize laser systems for our applications with research collaborators. To date, we have tested ablation of insulation and cutting of single or bundles of ultrafine microwires with three different laser systems: an excimer laser system, a 1W DPSS UV laser system and a laser microdissection system, the Leica LMD7. Figure 12 shows these results. Basically, all three laser systems were good enough to ablate the insulation of microwires. Even with the 1W UV laser which had the lowest power among the three, it was possible to cut the 12.7 μm Nichrome wire (see Figure 12D). We expect that having a laser system dedicated to insulation ablation on and cutting BMEPs would unleash many possibilities in BMEP designs in the near future.

### Combinatorics Uses of Multisite Wire Braids to Significantly Increase Yield per Wire

The braid plate design presented here was developed to support use of combinatoric signal separation from multisite wires organized using braid patterning. In principle, combinatoric methods allow very large numbers of unique spatial sites to be coded by the combination of wires present, and use of multisite wires. The wire mixes at each site determine the correlated signal signatures resulting from neurons at a specific spatial site in the multisite wire recording. In practice, we recognize that there must be limits to the possibilities here, set by information content of spikes and by collisions of spike events. Information separation techniques for analyzing spike train data have been used in various ways in the past (Brown et al., 2001; Snellings et al., 2006; Cui et al., 2008; Jackel et al., 2012; Hu and Liang, 2014). Our strategy tested here is to design wire arrangements that are consistent with the specific capabilities of infomax ICA to separate sources. These strategies support a more modest gain in multisite wire yield than employing the full combinatoric space. However, as shown here, these designs allow near full recovery of the spatial mixture at a given spatial site from the probe array as one of the 9 activations/ICs extracted by ICA. Nine wires with four sites per wire arranged as iterated 3 choose 2 arrangements provided full recovery of the neural mixture signal at each site. The recordings were the equivalent of 9 tetrodes but built from only 9 wires (as compared to 36 wires for 9 separate tetrodes). The potential neural yield was increased by a factor of 4. In the individual activation/IC at a given spatial site, individual simulated spikes were easily separated in Matlab or using an offline sorter after export to a.NEV file. However, in the single activation/IC analysis we found that collisions among neurons ***at a site*** caused errors. The collision rate at a spatial site was that of 4 Poisson processes (4 neurons) at 40 Hz within the spike capture window. As measured from the 10 simulated data trials of 36 unique spike trains at 40 Hz, the percent of spikes implicated in wire-level collisions averaged 62.75 ± 0.71, and the percent of spikes implicated in site-level collisions averaged 18.16 ± 1.20. Only the latter caused neuron isolation errors in our design. Higher spike rates have higher collision rates and more loss. Lower spike rates give higher fidelity recovery as collisions reduce. For example, we calculated that in the same _9_C_4_ arrangement, over 10 simulation trials with 36 spike trains at 5 Hz, the percent of spikes implicated in wire-level collisions decreased to an average of 11.93 ± 1.66, and the percent of spikes implicated in site-level collisions decreased to an averaged 2.47 ± 1.63. Thus these methods are better in systems with sparse low frequency activity. At the same time, as rates increase, under population methods, the lower fractional yield of well identified spikes still represented many more spikes than at lower rates, albeit flattening the frequency increase somewhat. The complete separation of spatial sites into the 9 activations, means that the local collisions may be mitigated and available for further analysis as tetrode data even at higher rates (Gray et al., 1995; Takahashi et al., 2003; Franke et al., 2010), although here we did not explore this directly. This feature of spatial site separations eliminated significant fractions of collisions directly. On a single 4 site multisite wire the collisions of 16 neurons were present, four times the final collision rate at the extracted spatial site. Our simulations support the high value of combinatoric braids for reducing the number of wires which are needed to achieve a given neural yield in invasive neural prosthetics. However, off-line signal processing or quite sophisticated recursive signal processing in real time is needed to fully leverage these gains. As further miniaturization and computing power gains proceed, the capacity for these signal processing features incorporated as part of device designs will become more feasible.

### Various Possible Applications of Figured Braid BMEPs and Combinatorics

#### Suture-Like BMEPs as an Intrafascicular Microelectrode

Unlike neural interfaces in CNS, peripheral nerve interfaces require much smaller size and more mechanical flexibility of electrode design due to the size, length, small diameter and freely moving condition without bone support of peripheral nerves. The most commonly used electrode types for peripheral nerve interfaces are cuff electrodes which have a C or U cuff-like shape to grab or wrap the exterior of peripheral nerve. These are also called extrafascicular electrodes. The FINE (flat interface nerve electrode) is also a modified cuff electrode in a rectangular shape to increase the contacting surface area and functional selectivity with multiple contacts (Tyler and Durand, 2002). For decades, the cuff electrode has proven to be a stable electrode type for clinical applications (Fisher et al., 2009; Polasek et al., 2009; Tan et al., 2014, 2015). Because there is no penetration into tissue, the extrafascicular electrodes do not directly damage the peripheral nerve, but exert a physical compression force to make a proper contact between the cuff and the nerve. However, disadvantages of extrafascicular electrodes include lower functional selectivity and requirement of higher electrical current level to achieve similar effects as can be evoked by intrafascicular electrodes. Intrafascicular probes are directly inserted into the peripheral nerve by penetration (Leventhal and Durand, 2003; Grinberg et al., 2008; Badia et al., 2011). Thus, though the intrafascicular electrode is a more invasive method than the extrafascicular, it has the significant advantage that it achieves higher selectivity with lower stimulation currents. The LIFE (longitudinal intra-fascicular electrode) (Yoshida and Horch, 1993; Dhillon et al., 2004), tf-LIFE (thin film LIFE) (Xavier et al., 2007; Kundu et al., 2014), DIME (distributed intrafascicular multi-electrode) (Thota et al., 2015), TIME (transverse intrafascicular multi-channel electrode) (Boretius et al., 2010) and USEA (Utah slanted electrode array) (Branner et al., 2001) are various intrafascicular electrodes that have been proposed and tested for peripheral nerve interfaces. The LIFE and DIME are based on a single 25 μm wire having one active site for recording/stimulation to insert the microwire longitudinally along with the nerve length and the difference between them is that the DIME is a coiled configuration system consisting of multiple LIFEs for safer and easier handling. The tf-LIFE was developed based on polyimide substrate to have 8 active sites and more mechanical flexibility, but it makes larger tissue damage than a single LIFE (folded film at the center, doubled 10 μm thick × 220 μm width with an 80 μm diameter tungsten needle for penetration). The TIME is also a modified tf-LIFE with 10 active sites to transversely penetrate the nerve instead of longitudinal penetration and it leaves a similar footprint as the tf-LIFE transversely in tissue. Though the USEA has very good selectivity, it makes larger and broader damage in the tissue due to its relatively larger scale. Electrode researchers have been exploring various ways to find a proper balance between less invasiveness and better performance for functional selectivity.

Currently we are assessing the potential of suture-like BMEPs as an intrafascicular microelectrode for peripheral nerve interfaces. As shown in Figure 10A,B, the BMEP fabrication is available over any size of sutures. We expect that it is possible to use this type of BMEP as use of a regular suture and so call it a suture-like BMEP. Among other intrafascicular electrodes described above, the LIFE having only one channel has the smallest footprint (37 μm = 25 μm diameter of bare wire + 2 × Teflon insulation thickness 6 μm in case of DIME (Thota et al., 2015). However, because a 75–80 μm diameter tungsten needle attached to one end of the LIFE is used to penetrate the nerve (Xavier et al., 2007; Thota et al., 2015), the diameter of initial nerve penetration and potential damage is 75–80 μm, not the total diameter size of insulated wire for the LIFE. If the BMEP on a 10-0 suture as shown in Figure 10A is inserted into the nerve, the diameter of initial damaged nerve would be 99 μm which is 19 μm larger than the tungsten needle, but it has 6 channels (6 wires) and a mechanically higher flexibility. We have never measured the lateral bending stiffness of BMEP with 6 × 9.6 μm wires and 10-0 suture, but we estimate that it would have roughly 5–10 times higher lateral flexibility than the single 25 μm diameter Plantinum/Iridium wire with 6 μm thickness of Teflon insulation (the calculated bending stiffness is 4.95 KPa mm^4^) based on our previous data (the measured bending stiffness of BMEP with 12 × 9.6 μm was 3.12 KPa mm^4^) and theoretical calculation (see Kim et al., 2013). Moreover, if an absorbable 10-0 suture, such as Ethicon VICRYL is used, only the BMEP would be left like an empty tubular braid in the nerve after a certain amount of time for complete dissolvement. This combination of highly mechanical flexibility and empty tubular braid structure may reduce immunoresponses in the nerve as we have seen from CNS histology results (Kim et al., 2019). For less initial invasiveness, BMEPs without any suture would be also a possible candidate, though we cannot yet confirm that the BMEP without any suture would withstand the rigors of PNS penetration without breakage. A loose BMEP with 6 × 9.6 μm wires without a 10-0 suture in Figure 10C has 66 μm outer diameter which is smaller than the 75 μm tungsten needle used for the LIFE. The thickness of polyimide insulation is about 3 μm over 12.7 μm Nichrome wire manufactured by Sandvik (see Figure 12D). Under the assumption that the thickness of polyimide insulation over 9.6 μm Nichrome wire is also 3 μm, the total outer diameter of a single polyimide insulated 9.6 μm wire would be 15.6 μm. Thus, the calculated outer diameter of BMEP with 6 × 2 × 9.6 μm Nichrome wires would be 62.4 μm (4 × 15.6 μm) because the theoretical outer diameter of BMEP without any core could be estimated by the thickness of stacked four wires. This calculated value is very close to the measured diameter, 66 μm. This diameter seems the smallest one we can make with polyimide insulated 9.6 μm Nichrome wires manufactured by Sandvik. In the example of a BMEP with 6 × 2 × 9.6 μm wires without any suture, its diameter is 98 μm which is almost same as the BMEP over the 10-0 suture in Figure 10A, but it has 12 channels. Furthermore, by manipulating positions and exposed length of active sites on the suture-like BMEP, the BMEP with multiple channels could be also used longitudinally or transversely. A currently unknown question is “which design is better for less chronic immunoresponeses in the peripheral nerve? Is a smaller diameter of BMEP with 6 × 9.6 μm wires always better? Or is a slightly larger diameter version of the same BMEP with 6 × 9.6 μm wires better due to the larger empty spaces and diffusion channels in the tubular braid lattice structure?”. Depending on this answer, we can try different strategies, such as the same BMEP over 10-0 (Figure 10A) vs. 5-0 (Figure 10B), the BMEP with 6 × 2 × 9.6 μm wires (Figure 10E) vs. 12 × 9.6 μm wires (Figure 10F), etc.

In summary, the suture-like BMEPs are a very promising design as an intrafascicular electrode for peripheral nerve interfaces because they provide at least 6 channels, higher mechanical flexibility than the single wire for the LIFE (which has the smallest outer diameter among intrafascicular electrodes proposed thus far), and likely also the same initial nerve damage as the LIFE. In addition, with the suture-like BMEPs either longitudinal or transvers penetration is possible with active site manipulation depending on applications.

#### Combination Applications With BMEPs

Beside the suture-like BMEP and modified geometry of BMEPs for combinatorics, we can also propose various other ideas for future possible applications of BMEPs. Currently available motorized robotic arms as a prosthetic arm for amputees utilize electromyograph (EMG) signals from muscles on a remained portion of arm or combination of muscles on chest, shoulder, and back to control the movements of bionic arm. For implanted EMG recordings, the BMEP with 6 × 9.6 um wires or more without any core, as shown in Figure 10C, may in future have potential as an implantable EMG electrode.

BMEPs over an optical fiber or GRIN lens would be ideal for an application requiring both functions of light emission for optogenetics and optical neural recording and also electrophysiology for fast recording or stimulation. GRIN lens based braids are a possibility that we are testing directly. BMEPs over a glass micropipette may be ideal for an application requiring drug delivery and electrophysiology at the same target position. Due to the freedom of length limitation, very long BMEPs might be used for deep brain stimulation (DBS) applications or recording in DBS closed loop applications. If conventional DBS probes in modified configuration are used as the core inside the BMEP, the core could be used conventionally as the initial recording and stimulating electrode to verify and find the target population in a deep brain area. However, in this case, once the target was found, the recording functions from the BMEP along the insertion track could be used in feedback modulation applications, without changing the position of the BMEP. Maybe in future applications only BMEP assemblies would be left in place as both the stimulating electrodes, recording electrodes and leads in the target population and electrode track, reducing implant footprints. There are clearly many opportunities.

The potential and promise of BMEPs, is as a design space for probe construction in 3D topologies which can be extended to many different designs, signal separation methods, and applications as needed for different target scientific or clinical purposes.

## Ethics Statement

Experimental procedures were approved by Drexel University Institutional Animal Care and Use Committee.

## Author Contributions

TK, KS, JW, HL, and SG: conceptualization. TK, KS, and SG: methodology. TK, KS, CD, JW, HL, and SG: validation. TK, KS, and SG: formal analysis. TK, KS, and SG: investigation. HL and SG: resources. TK, KS, CD, JW, HL, and SG: data curation. TK, KS, and SG: writing (original draft preparation). TK, KS, CD, JW, HL, and SG: writing (review and editing). TK, KS, CD, and SG: visualization. HL and SG: supervision. SG: project administration. HL and SG: funding acquisition.

## Conflict of Interest Statement

The authors declare that the research was conducted in the absence of any commercial or financial relationships that could be construed as a potential conflict of interest.
